# Prevalence of Carpal Tunnel Syndrome (CTS) among medical laboratory staff at King Saud University Hospitals, KSA

**DOI:** 10.1186/1753-6561-9-S1-A55

**Published:** 2015-01-14

**Authors:** AM Bardeesi, AA Al-Twair, AA Al-Mubarek

**Affiliations:** 1 The Department of Family and Community Medicine, College of Medicine, King Saud University, Kingdom of Saudi Arabia

## Background

Carpal tunnel syndrome (CTS) is a group of symptoms resulting from local compression of the median nerve at the wrist leading to subsequent functional impairment and local ischemia of the median nerve. Occupations that involve repetitive hand movements carry a great risk for developing CTS and laboratory occupations fall under this category. We decided to investigate in this topic due to its absence in the Saudi health literature. The aim of this study was to determine the prevalence of CTS in the laboratory workers of KSU hospitals by using self-administered Boston Carpal Tunnel Questionnaire. It was also intended to determine the most commonly reported symptoms, the important independent risk variables included in the development of CTS including age, sex, BMI, years of employment, work pattern, and working hours per week.

## Methods

This is a quantitative observational cross-sectional study that was held in KSU hospitals’ laboratories with a total of 225 participants. a standardized questionnaire known as " Boston Carpal Tunnel Questionnaire (BCTQ)" was used for the assessment of symptoms severity and functional status in carpal tunnel syndrome. Data Analysis was made by IBM SPSS Statistics software version 21.0. For data interpretation, the total scores were classified into groups (mild, moderate & severe) using percentiles, and Chi-square test was used to observe the association between study categories and the independent outcome variables. The means were also compared using student’s t-test for independent samples.

## Results

Out of the 225 participants, 57 were found to be severely symptomatic. The prevalence rate of the severely symptomatic participants was determined as 25.3%. Among the severely affected participants, females were more than males (58% > 42%) and the difference was statistically significant (p=0.045). Technicians affected (91.2) were more than attendants (8.8%) and the difference was statistically significant (p=0.042). There was statistically significant association between the dominant and affected hand (p<0.0001). Wrist pain was the leading reported symptom (85.2%).

## Conclusions

The prevalence rate of CTS in KSU hospitals’ staff (25.3%) was close to was found in literature (21.5%). So, laboratory workers are at risk of developing CTS, especially females and technicians, with the dominant hand most likely to be affected. If decent educational and preventive efforts are not considered towards this population, further deterioration is expected to those already affected and newer cases will appear.

